# Privacy Preserved and Secured Reliable Routing Protocol for Wireless Mesh Networks

**DOI:** 10.1155/2015/636590

**Published:** 2015-09-21

**Authors:** Navamani Thandava Meganathan, Yogesh Palanichamy

**Affiliations:** ^1^Department of Computer Science and Engineering, Easwari Engineering College, Ramapuram, Chennai, Tamilnadu 600 089, India; ^2^Department of Information Science and Technology, College of Engineering, Anna University, Guindy, Chennai, Tamilnadu 600 025, India

## Abstract

Privacy preservation and security provision against internal attacks in wireless mesh networks (WMNs) are more demanding than in wired networks due to the open nature and mobility of certain nodes in the network. Several schemes have been proposed to preserve privacy and provide security in WMNs. To provide complete privacy protection in WMNs, the properties of unobservability, unlinkability, and anonymity are to be ensured during route discovery. These properties can be achieved by implementing group signature and ID-based encryption schemes during route discovery. Due to the characteristics of WMNs, it is more vulnerable to many network layer attacks. Hence, a strong protection is needed to avoid these attacks and this can be achieved by introducing a new Cross-Layer and Subject Logic based Dynamic Reputation (CLSL-DR) mechanism during route discovery. In this paper, we propose a new Privacy preserved and Secured Reliable Routing (PSRR) protocol for WMNs. This protocol incorporates group signature, ID-based encryption schemes, and CLSL-DR mechanism to ensure strong privacy, security, and reliability in WMNs. Simulation results prove this by showing better performance in terms of most of the chosen parameters than the existing protocols.

## 1. Introduction

Wireless mesh networks (WMNs) are an emerging wireless technology by having more advantages over other wireless ad hoc networks. WMNs comprise two types of nodes: mesh routers and mesh clients. Mesh routers, which are static and power-enabled, form a wireless backbone of the WMNs and interwork with the wired networks to provide multihop wireless Internet connectivity to the mesh clients. Mesh routers form a mesh backbone through which mesh clients can access the network. Unlike mesh routers, the mesh clients can be battery-operated mobile nodes. In mesh networks, mesh routers can also directly mesh with each other [1]. WMNs are of three types. They are (i) infrastructure based WMN, (ii) hybrid WMN, and (iii) client WMN. Infrastructure based WMN has a mesh backbone which consists of mesh routers and gateways. Client nodes can connect to the backbone through mesh routers.

The properties pertaining to WMNs like open shared medium, absence of centralized server, and dynamic topology make the network be vulnerable against adversaries (malicious nodes) from both inside and outside. In general, the internal malicious nodes are more difficult to detect in wireless networks than the external attacker nodes. A number of secure routing mechanisms using public key cryptographic mechanisms are discussed in the literature. However, relying on public key cryptosystems alone does not provide complete security against the internal attacks. To enforce security against malicious nodes, several reputation mechanisms are proposed in the existing systems to monitor the behavior of the neighbor nodes and also for evaluating the reputation metrics of the neighbor nodes. However, these mechanisms are not sufficient to provide complete privacy protection and also to ensure better link reliability in WMN. In most of the situations, the communication in WMNs has different kinds of sensitive user information which have to be protected and secured from the unauthorized nodes like malicious nodes. Hence, strong privacy protection mechanisms and secure routing mechanisms are needed to protect communication that involves sensitive information in WMNs.

Thus, it is analyzed that privacy and security issues are the vital problems in the design of WMNs. The clients should have end-to-end security and privacy assurance [2]. Several privacy preserved secure routing schemes proposed for WMNs have been discussed in the literature. However these schemes do not provide complete privacy protection. Moreover they do not ensure better link reliability that is required to select the optimal route in infrastructure based WMNs. In [3, 4], the authors have proposed reputation evaluation mechanisms to enforce security and to defend against internal attacks in WMNs. In these reputation evaluation mechanisms, the reputation computation incorporates traditional weighted average model to compute the link quality metric which in turn evaluates the direct behavior of the nodes. However, in general, wireless environment needs cross-layer based routing metrics to guarantee the accurate measurement of link quality. Hence, in this work we employ cross-layer design in reputation evaluation mechanism to enhance the security of WMNs.

Several routing metrics designed for capturing the link quality to discover high performance routing paths in wireless mesh networks have been discussed in [5, 6]. However, these routing metrics are designed by having strong assumption that all nodes behave honestly during the forwarding of packets and the network is more reliable. This assumption may not be true always for infrastructure based WMNs, where all the nodes do not cooperate all the times and link reliability is also not guaranteed. Unlike the complex infrastructure of cellular networks like base station and mobile switching centre, the WMN infrastructure like mesh router could be relatively easily reached and modified by attackers. The attackers can be able to track the packets in the network and can fetch the information from the packets. By reviewing the existing works, it is known that there is no optimal routing solution that is capable of doing all the three following operations in WMNs: preserving privacy, providing security against adversaries, and discovering high performance reliable routing path. Hence, in order to establish secured and reliable routing path with better privacy protection, the existing routing protocols have to be enhanced further in terms of security and privacy to defend against various DoS attacks.

In this paper, Privacy preserved and Secured Reliable Routing (PSRR) for infrastructure based wireless mesh networks is proposed to provide maximum privacy protection and to have maximum security against adversaries. The proposed protocol also provides better network performance by selecting the more reliable route. Privacy protection in WMNs can be achieved by implementing group signature and ID-based encryption scheme. The proposed protocol protects the network against packet dropping and misdirecting attacks by implementing a new Cross-Layer and Subject Logic based Dynamic Reputation (CLSL-DR) mechanism in mesh routers. It also provides optimal path for data transmission by selecting secured reliable path during route discovery. The major contributions of this paper comprise (1) design of a new Privacy preserved Secure Reliable Routing protocol for WMNs, (2) protection against packet dropping and misdirecting attacks by introducing a novel Cross-Layer and Subject Logic based Dynamic Reputation (CLSL-DR) mechanism, (3) minimizing the control packets overhead by means of trust level (TL) metric, and (4) optimal path discovery for efficient data transmission by selecting a secured reliable path using cross-layer information exchange.

The rest of this paper is structured as follows.  Section 2 discusses related works on privacy, security, and reputation mechanisms for wireless mesh networks. In Section 3, we present the proposed protocol. Security and privacy analyses are discussed in Section 4.  Section 5 discusses implementation and performance analysis in terms of the simulation results. Finally, in Section 6, we conclude the paper with future enhancements.

## 2. Related Work

In recent years, more numbers of secure routing protocols with privacy protection have been proposed for WMNs. These routing protocols consider privacy and security in different levels to improve the network performance. To ensure whether the packets are forwarded correctly and to provide complete privacy preservation to the user traffic messages, secure reliable routing protocol with better privacy protection in WMNs is needed.

Mahmoud et al. [7] proposed a low-overhead secure privacy preserved routing protocol in hybrid ad hoc networks in which symmetric-key-cryptography operations and payment system are used to develop secure privacy preserved route discovery and data transmission. However, this protocol will not be applied for WMNs where there is no centralized server to process the payment receipts. In our previous work [8], we have proposed a routing scheme that gives better protection against security attacks and anonymity in wireless mesh networks. We introduced secured identity based routing scheme to provide security against attacks and anonymity. However, it does not provide complete privacy protection for data packets and control packets. Mahmoud et al. [9] developed a stable and reliable routing protocol named E-STAR in heterogeneous multihop wireless networks. This protocol combines payment and trust systems with trust based and energy aware routing protocols. Multidimensional trust values are used for computation of trust and reliability in routing. Payment systems are used for rewarding the nodes which are forwarding the packets. However, the protocol incurs more overhead in terms of processing payment receipts and trust metrics evaluation. Wan et al. [10] proposed an unobservable secure on demand routing protocol for mobile ad hoc networks to provide complete privacy protection by satisfying the privacy requirements such as anonymity, unlinkability, and unobservability. In this protocol, group signature and ID-based encryption techniques are implemented along with the route discovery protocol to satisfy the above requirements in the network. However, the protocol does not address wormhole attacks and other DoS attacks. Paris et al. [11] proposed a novel cross-layer based routing metric, named Expected Forwarding Counter (EFW), to defend against packet dropping attacks. This metric considers link quality of wireless links using Medium Access Control (MAC) layer measurements and also monitors the forwarding behavior in network layer to select secure reliable routing path in WMN. Two further variants of EFW named Minimum Expected Forwarding Counter (MEFW) and Joint Expected Forwarding Counter (JEFW) are also proposed in the same paper to solve the problem of packet dropping behaviour of selfish nodes. Among these metrics, MEFW is proved as a robust link quality metric to select secure reliable routing path in WMN. Khan et al. [12] designed a secure routing protocol for infrastructure based wireless mesh networks. In this scheme, a new routing metric called Unreliable Value (UV) is proposed, which is capable of searching the shortest secure path by computing UV of the neighbors by implementing a two-hop passive acknowledgment scheme. However, this protocol is more vulnerable to packet modification and tampering attacks, since authentication and encryption mechanisms are not implemented in the route discovery algorithm.

Khan et al. [13] introduced a secure route selection scheme in wireless mesh networks. This scheme is based on two-hop passive acknowledgement mechanism which is used to prevent the network from packet dropping attacks. However, this mechanism has not provided complete security solution against all types of packet dropping attacks. In [14], Bansal et al. introduced a secure routing protocol for hybrid wireless mesh network which uses cryptographic extensions to provide authenticity and integrity of Hybrid Wireless Mesh Protocol (HWMP) routing messages and prevents unauthorized manipulation of mutable fields in the routing information elements. This protocol suffers from routing acquisition delay and control packet overhead because of cryptographic extensions during route discovery. And also, this protocol is more vulnerable to attacks caused by colluding compromised nodes within the network. You and Wang [15] proposed an efficient secure routing protocol for hybrid wireless mesh network. The protocol implements several cross-layer parameters to select an optimal route based on security and robust against various multihop threats in WMNs. Khan and Loo [16] proposed a Cross-layer Secure and Resource aware On demand Routing (CSROR) protocol for hybrid WMN to ensure routing security and fulfill different applications' specific requirements for multimedia delivery and real-time communication. The protocol selects an optimal route by considering routing security as well as different cross-layer parameters. It is resilient to different packet dropping attacks, but the protocol is not suitable for the network with nodes having high mobility and also not providing solution for packet modification attacks. Seth and Gankotiya [17] discussed various Denial of Service (Dos) attacks and detection methods in WMNs. They had discussed these attack detection methods in physical layer, MAC layer, and routing layer and concluded that cross-layer design is the better solution to detect these attacks in routing layer.

Yu et al. [18] have proposed a new dynamic hierarchical reputation evaluation scheme to provide secure solution against intruders for hybrid wireless mesh networks. This scheme is based on virtual cluster structure and behavior, correlations of the nodes in the network. However, this scheme does not address link reliability to provide high performance routing path. Sun et al. [19] proposed a security architecture to ensure unconditional anonymity for honest users and to achieve traceability of misbehaving users in the network. They have implemented ticket based protocols to resolve the conflicting requirements of privacy and security. Kathyaini and Ananthakumaran [20] proposed a protocol which implements quantum principles in wireless mesh network to achieve anonymity and security. Even though the protocol provides better authentication, it does not address all types of network layer attacks.

After reviewing the previous work, we propose a new scheme for routing in wireless mesh networks to provide better privacy and security and also to select an optimal path for data transmission. This can be achieved by implementing group signature and ID-based encryption mechanisms, Cross-Layer and Subject Logic based Dynamic Reputation (CLSL-DR) mechanism along with the route discovery protocol.

## 3. Privacy Preserved Secure Reliable Routing Scheme

In this section, we explain the Privacy preserved Secured and Reliable Routing (PSRR) scheme for wireless mesh networks. The following subsections describe the functions of the proposed protocol in detail. This scheme provides complete privacy protection to preserve privacy and implements CLSL-DR mechanism to defend against the internal attacks caused by compromised nodes of WMN. As a result, the discovered path is able to provide reliable communication between the source and the destination.

### 3.1. System Model

#### 3.1.1. Network Model and Assumptions

We consider an infrastructure based wireless mesh network which consists of mesh routers and mesh clients. Multiple mesh routers communicate among themselves to form a wireless backbone that forwards the user traffic to gateways. Mesh clients can directly connect to the nearest mesh router. We further assume that the traffic from the source client node to the destination client node passes through the routers present in the mesh backbone. We combine group signature scheme and ID-based encryption scheme [10, 21] to provide complete privacy protection in WMNs. Both group signature and ID-based encryption schemes are based on elliptic curve groups with the order of a big prime number. In accordance with the group signature scheme, an offline key server generates group public key gpk, which is known to all other nodes, and group signature key gsk for each node in the network. For implementing ID-based encryption scheme, two elliptic curve groups *G*1 and *G*2 with order of a big prime number *p* are considered to generate private and public keys. A bilinear pairing map “*e*” is also defined as *G*1 × *G*1 → *G*2 according to the above-discussed works. Initially the key server generates master secret key *s* and ID-based private key for every node *N* as *K*_ID_*N*__ = *s* · *H*_1_(ID_*N*_), where *H*_1_(ID_*N*_) is the secure one-way hash function which maps the identity of node *N* to an element in group *G*1. The corresponding public key is also generated for the node *N* as (*p*, *G*1, *G*2, *e*, *R*, *R*_pub_, and  *H*_1_), where *R* is a random generator, which is selected by the key server, and *R*_pub_ is computed as *s* · *R*.

#### 3.1.2. Attack Model

We assume that an attacker is capable of monitoring the entire traffic passing through the network. The attackers may attempt to eavesdrop all the network communication and analyze them to obtain information about the participants, packet type, and so forth. Assumption also includes the possibility of adversaries launching active attacks by injecting, modifying, and dropping packets within the network. Some of the internal nodes may be compromised by the attackers to generate any Denial of Service attack in the network.

#### 3.1.3. Notations

The symbols and their description that are used in the proposed scheme are given in the Notations section. The symbol *H*_*i*_(*∗*) represents the three levels of secure one-way hash functions with *i* = 1,2, 3. These functions are used during key establishment and route discovery process. *H*_1_ function maps the identity of node *N* to an element in *G*1, *H*_2_ function maps an element in *G*1 to a session key, and *H*_3_ function maps a session key with a random pseudonym.

### 3.2. The Proposed Scheme PSRR

**Figure 1:**
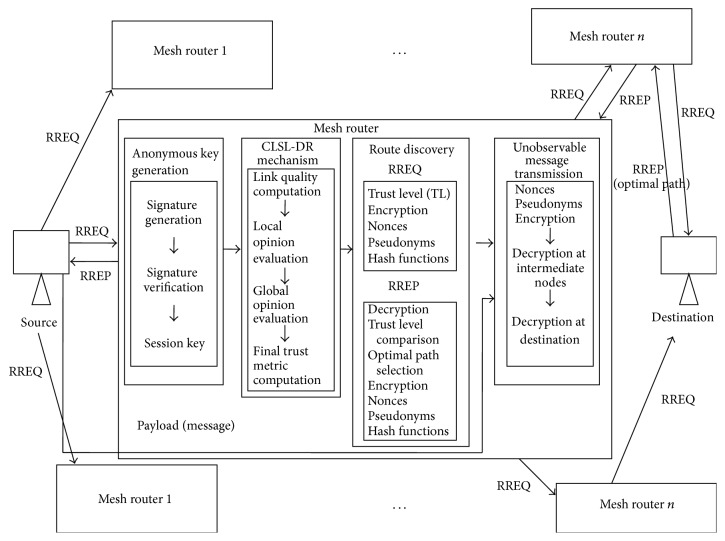
Functional components of PSRR.

The proposed scheme includes the following phases: anonymous key generation, CLSL-DR mechanism, route discovery, and unobservable message transmission. The functional components of the proposed PSRR protocol are shown in Figure 1. Each node present in the network shares a session key between the neighbor nodes and establishes a local broadcast key by anonymous key establishment algorithm. According to the proposed scheme, the discovered routing path is privacy preserved, secured, and reliable. During route discovery, CLSL-DR mechanism is invoked on the mesh routers to compute cross-layer based reputation to isolate the malicious nodes in the routing path. In the route discovery process, source node forwards an unobservable Route Request message based on the trust level (TL) metric to minimize the control packets overhead and the request reaches the destination through the intermediate nodes. This metric is appended and cumulatively added in the RREQ packet until it reaches the destination. Since the source node broadcasts the Route Request, the destination receives several route requests until the time period *T* through different paths. It selects an optimal path as the most trusted path for data transmission. Then the destination performs a unicast Route Reply to the source through the discovered route. Finally the packets are transmitted from source and reach the destination through this path. Following subsections describe the functions of each module in detail.

#### 3.2.1. Anonymous Key Generation

In this phase, each node in the network establishes a session key with each of its neighbors within its radio range. Assume node *S* has a private signing key gsk_*S*_ and private ID-based key *K*_ID_*S*__ in the wireless mesh network. The following algorithm describes the step by step procedure of anonymous key generation process between source node (*S*) and neighbor node (*Ij*) with respect to the Notations section.

(1) Source node *S* generates the random number *N*_*S*_ and computes the signature as SIG gsk_*S*_(*N*_*S*_ *R*) using its group signature key, where *R* is the generator of group *G*. *S* broadcasts the computed signature to its neighbors.
(2) Now intermediate node *Ij* receives the message from *S* and verifies signature sent by *S*. If verification is successful, then the intermediate node generates random number (*N*_*Ij*_ *R*) and computes the signature  SIG gsk_*Ij*_(*N*_*S*_ *R*∣*N*_*Ij*_ *R*).
(3) Intermediate node *Ij* also creates shared session key *K*_*SIj*_ = *H*_2_(*N*_*S*_ *N*_*Ij*_ *R*) and replies with {*N*_*Ij*_ *R*, $SIG\;{gsk}_{Ij}\left(N_{S}R|N_{Ij}R\right)E_{K_{SIj}}({\overset{-}{K}}_{Ij}|N_{S}R|N_{Ij}R)\}$ to *S*, where ${\overset{-}{K}}_{Ij}$ is the local broadcast key of *Ij*.
(4) Source node *S* again verifies the signature, if it is successful, and then it computes session key between *Ij* and itself as *K*_*SIj*_ = *H*_2_(*N*_*S*_ *N*_*Ij*_ *R*). Then it generates local broadcast key ${\overset{-}{K}}_{S^{*}}$ and sends $E_{K_{SIj}}({\overset{-}{K}}_{S^{*}}|{\overset{-}{K}}_{Ij^{*}}|N_{S}R|N_{Ij}R)$ to *Ij*.
(5) Finally intermediate node *Ij* receives the message from *S* and computes same session key as *K*_*SIj*_ = *H*_2_(*N*_*S*_ *N*_*Ij*_ *R*). It decrypts the message received from *S* to get the local broadcast key of  *S* $({\overset{-}{K}}_{S^{*}})\cdot {Nym}_{N}$.

This process is repeated for each of the intermediate nodes to get the shared session key with its neighbors. This key establishment process is implemented based on an efficient security scheme such as Elliptic Curve Diffie Hellman (ECDH) key exchange and group signature scheme which has more advantages such as more security strength and faster, better performance, compared to other cryptographic schemes [21]. At the end of this key generation process, all intermediate nodes have their own shared session key with their neighbor nodes which are used during route discovery process and these keys are generated anonymously without knowing each other.

#### 3.2.2. Cross-Layer and Subject Logic Based Reputation Scheme

Here, we propose a new cross-layer and subject logic based reputation mechanism which is an improved version of the scheme presented in [3, 4]. In this mechanism, cross-layer based metric and uncertainty are incorporated in association with subject logic into the reputation computation algorithm to detect and isolate the malicious nodes and thereby finding reliable routing paths. The metric considers network layer observations of forwarding behavior in combination with MAC-layer measurements of wireless link quality to select more reliable and high performance path. According to subject logic, a trust metric is represented as an opinion to express subjective beliefs. Each opinion is defined by four parameters and it is specified as *O*_*m*:*n*_ = (*T*_*m*:*n*_, *D*_*m*:*n*_, *U*_*m*:*n*_, *R*_*m*:*n*_), where *T*_*m*:*n*_ represents node *m*'s trust on node *n*, *D*_*m*:*n*_ represents node *m*'s distrust on node *n*, *U*_*m*:*n*_ represents node *m*'s uncertainty on node *n*, and *R*_*m*:*n*_ is the base rate of *m*'s trust on node *n*. These parameters should satisfy the following conditions:

(1) $$\begin{array}{c} T_{m:n}+D_{m:n}+U_{m:n}=1.0, \end{array}$$

where *T*_*m*:*n*_, *D*_*m*:*n*_, *U*_*m*:*n*_, and *R*_*m*:*n*_ ∈ [0.0,1.0].

By assuming the opinion as a decision, the final trust metric is computed as

(2) $$\begin{array}{c} F\left(\;O_{m:n}\;\right)=T_{m:n}+R_{m:n}U_{m:n}. \end{array}$$

*(1) Reputation Computation*

*Local Opinion.* Let us assume that *m* and *n* are the two neighboring nodes. The final opinion of node *m* to *n* *O*_*m*:*n*_^final^ is computed by having both local observation (local opinion) and global observation (global opinion):

(3) $$\begin{array}{c} O_{m:n}^{final}=\left\{\;O_{m:n}^{loc},O_{m:n}^{glo}\;\right\}. \end{array}$$

The local opinion of node *m* to node *n* *O*_*m*:*n*_^loc^ = *T*_*m*:*n*_^loc^, *D*_*m*:*n*_^loc^, *U*_*m*:*n*_^loc^, and *R*_*m*:*n*_^loc^ is computed and it is stored in *m*'s local reputation table with respective node's ID and the values are computed as follows:

(4) Tm:nloc=STm→nNTm→n∗LQm→n,Dm:nloc=NFm→nNTm→n∗LQm→n,Um:nloc=1.0−Tm:nloc−Dm:nloc,

where ST_*m*→*n*_ represents the number of packets received from *m* and successfully forwarded by *n*, NF_*m*→*n*_ represents the number of packets received from *m* and not forwarded by *n*, and NT_*m*→*n*_ is the total number of packet transmissions received from *m*. LQ_*m*→*n*_ denotes the link quality metric from *m* to *n*, which is computed as in (5).

To measure link quality in WMNs, we use a novel cross-layer based reliable routing metric named Minimum Expected Forwarding Counter (MEFW) [11] to isolate the malicious or selfish nodes during route discovery. MEFW considers worst dropping behavior of the nodes and it is more robust against packet dropping attacks. Compared to the traditional mechanisms for estimating the link quality in WMNs, MEFW metric simplifies the network representation and selects the most reliable and high performance routing path by considering routing layer observations of forwarding behavior as well as MAC-layer measurements of wireless link quality:

(5) LQm→nMEFWm→n=MEFWn→m=11−Pmn1−Pnm·11−max⁡Pd,mn,Pd,nm,

where *P*_*mn*_ and *P*_*nm*_ denote the packet loss probability of the wireless link (*m*, *n*) in forward and reverse directions, respectively. *P*_*d*,*mn*_ and *P*_*d*,*nm*_ represent the dropping probabilities in *m* to *n* direction and *n* to *m* direction, respectively, at the network layer of node *n*. It is possible to discover high reliability paths that are able to provide better Packet Delivery Ratio with the help of this metric since this metric is able to decide the quality of the links. Whenever a new node joins the network, the default trust opinion for the new node is set by the other nodes as (0.0,0.0,1.0, *R*). These local opinions are updated in the local reputation table periodically from time to time.

*Global Opinion.* These are useful when the local opinions are not enough to judge a node. If a node *m* wants to collect global opinions on node *n* from their common neighbor nodes, it just passes the reputation query to them. When node *m* receives global opinions on node *n* from two recommenders, and if their opinions conflict with each other, then *m* has to decide which recommender node is more trustworthy and get the opinion from that node and discard the opinion from the other node. For more than two recommenders, let us assume that *R* is the set of recommenders, and for each recommender *i* ∈ *R*, a unique weight is assigned and it is calculated according to (6):

(6) $$\begin{array}{c} w_{i}=\frac{F\left(O_{m:i}\right)}{\sum_{k\in R}F\left(O_{m:k}\right)}, \end{array}$$

where

(7) $$\begin{array}{c} F\left(\;O_{m:i}\;\right)=T_{m:i}+R_{m:i}U_{m:i}. \end{array}$$

Now, the global opinion *O*_*m*:*n*_^glo^ = (*T*_*m*:*n*_^glo^, *D*_*m*:*n*_^glo^, *U*_*m*:*n*_^glo^, *R*_*m*:*n*_^glo^) is calculated as shown in

(8) Tm:nglo=∑k∈RwkTk:nloc,Dm:nglo=∑k∈RwkDk:nloc,Um:nglo=∑k∈RwkUk:nloc,Rm:nglo=∑k∈RwkRK:nloc.

After obtaining the local opinion and the global opinion, a final opinion *F*_*m*:*n*_^final^ = (*T*_*m*:*n*_^final^, *D*_*m*:*n*_^final^, *U*_*m*:*n*_^final^, *R*_*m*:*n*_^final^) is calculated as shown in

(9) Tm:nfinal=Tm:ndir·Um:nglo+Tm:nglo·Um:ndirUm:ndir+Um:nglo−Um:nglo·Um:ndir,Dm:nfinal=Dm:ndir·Um:nglo+Dm:nglo·Um:ndirUm:ndir+Um:nglo−Um:nglo·Um:ndir,Um:nfinal=Um:ndir·Um:ngloUm:ndir+Um:nglo−Um:nglo·Um:ndir,Rm:nfinal=Rm:ndir·Um:nglo+Rm:nglo·Um:ndirUm:ndir+Um:nglo−Um:nglo·Um:ndir.

Since all the trust parameters will change over time, the trust relationship between any two nodes will also change dynamically. Whenever a new observation comes in, each node updates its trust table and the final trust is calculated by using a moving average model as shown in

(10) $$\begin{array}{c} F_{t1}=\alpha F_{t0}+\left(\;1-\alpha \;\right)F_{t1}, \end{array}$$

where *α*  (0 < *α* < 1) is the weighting factor which is used as normalizing factor between previous measurement and current measurement. The route discovery process uses this trust metric for selecting the secure routing path from source to destination.

#### 3.2.3. Route Discovery

Here we discuss the route discovery procedure of the proposed protocol based on the keys established and the computed trust level between the neighbor nodes. Assume that source node  *S* wants to find a route to destination node *D*. The route discovery process is carried out by Route Request (RREQ) and Route Reply (RREP) packets. The generation of RREQ and RREP packets is discussed as follows.

*(1) Route Request (RREQ).* Source node *S* generates a random number *N*_*S*_ *R* and a sequence number (snum) to uniquely identify the Route Request and also generates a nonce (Nonce_*S*_) to achieve unobservability. Then it computes the pseudonym as ${Nym}_{S}=H_{3}\left({\overset{-}{K}}_{S^{*}}|{Nonce}_{S}\right)$. Other route discovery parameters such as timestamp (ts) which specifies the time required to complete the route discovery and maximum number of intermediate hops required (max⁡*h*) are also appended within the RREQ message to reduce the route discovery time. To ensure anonymity and unobservability, *S* encrypts the identity information of source and destination nodes along with the random number using the identity of node *D* to create the cipher text as *E*_ID_*D*__  (ID_*S*_, ID_*D*_, *N*_*S*_ *R*). It means that only the destination node can decrypt the relevant cipher text by using its private ID-based key. Now, *S* applies one more level of encryption along with the route discovery parameters using the local broadcast key *K*_*S*^*∗*^_ to obtain $E_{{\overset{-}{K}}_{S^{*}}}\left(RREQ,TL,ts,\max h,E_{{ID}_{D}}\left({ID}_{S},{ID}_{D},N_{S}R\right)\right)$. The final RREQ message is formulated as shown in

(11) RREQ=NonceS,NymS,EK−S∗RREQ,TL,ts,max⁡h,EIDDIDS,IDD,NSR,snum.

Before broadcasting RREQ, source  *S* applies CLSL-DR mechanism to compute the reputation values of its neighbors according to the following steps.

(1) Source  *S* checks its local reputation table to retrieve local opinion *O*_*S*:*Ij*_^loc^ of a neighbor node *Ij* and also computes the final trust metric *F*(*O*_*S*:*Ij*_^loc^).
(2) If *F*(*O*_*S*:*Ij*_^loc^) ≥ *θ*, then *Ij* is considered as trustworthy node; else if *F*(*O*_*S*:*Ij*_^loc^) < *θ*, then *S* tries to collect the global opinions from the common neighbor nodes with *Ij*, where *θ* is the threshold parameter which lies between [0.0,1.0].
(3) To retrieve the global opinion, *S* forwards the Rep_query message to the common neighbor nodes and waits for the time period *T*.
(4) If a node's uncertainty opinion on its neighbor node *Ij* is less than 1, then it forwards its local opinion on *Ij* to *S*.
(5) When the time period *T* is ended, *S* collects all the global opinions from common neighbor nodes and assigns unique weight to them.
(6) Then *S* computes *O*_*s*:*Ij*_^glo^ and evaluates the final trust metric *F*(*O*_*S*:*Ij*_^glo^). If *F*(*O*_*S*:*Ij*_^glo^) ≥ *θ*, then *Ij* is considered as trustworthy node; otherwise, *Ij* is considered as malicious node and this state is recorded in the trust table.

According to the above steps, *S* evaluates the trust metric of its neighbor nodes. Now *S* forwards the anonymous RREQ message only to its neighbors whose trust metric is greater than *θ* as shown in (11). Let us assume that *Ij* is a trustworthy node and receives the RREQ message from *S*. Now *Ij* tries all its session keys shared with all its neighbors to find out ${\overset{-}{K}}_{S^{*}}$ by satisfying ${Nym}_{S}=H_{3}\left({\overset{-}{K}}_{S^{*}}|{Nonce}_{S}\right)$. If it is found, then it uses the key to decrypt the cipher text. Now it can find that it is a Route Request packet. TL is the trust level metric appended by the previous intermediate node. The total time required for route discovery is specified as ts and max⁡*h* denotes the maximum number of intermediate nodes required in the routing path. Node *Ij* also tries its private ID-based key to decrypt the inner message *E*_ID_*D*__(ID_*S*_, ID_*D*_, *N*_*S*_ *R*). If this cannot be decrypted, then *Ij* is not the destination node. Then it generates a new nonce Nonce_*Ij*_ and computes Nym_*Ij*_. The snum stored in the RREQ message is recorded in the intermediate node's routing table to identify duplicate RREQ messages. Now *Ij* also applies CLSL-DR mechanism to compute the reputation values of its neighbors according to the above steps. Once *Ij* receives the trust metric of all its neighbor nodes, it forwards the updated RREQ message according to (12) only to the selected neighbors whose trust metric is greater than *θ*. By selectively forwarding the RREQ packets, the control packets overhead are minimized. The trust level (TL) metric in the updated RREQ message is updated by cumulatively adding the existing value with the metric computed by *Ij* with each of its neighbor nodes:

(12) RREQ=NonceIj,NymIj,EK−Ij∗RREQ,TL,ts,max⁡h,EIDDIDS,IDD,NSR,snum.

In this manner, the other intermediate nodes repeat the above process to forward the RREQ message to its neighbor nodes. In this way, RREQ packet in its final form reaches the destination node. Usually multiple RREQ packets reach the destination for the same source.

*(2) Route Reply (RREP). *Destination node receives many RREQ packets from the same source. The destination node also tries its session keys to find out the local broadcast key to decrypt the cipher text. Once it is successfully decrypted, it tries its private ID-based key to decrypt *E*_ID_*D*__(ID_*S*_, ID_*D*_, *N*_*S*_ *R*). If it is decrypted then it knows that it is the destination node. By checking the snum field in the RREQ packets, it can receive same RREQ message from different neighbor nodes. To select privacy preserved secure reliable path, it waits for time period *T*. Once *T* is over, it compares the TL metric available in the received RREQ messages. The RREQ message which has the highest TL metric is chosen as the selected optimal path and RREP message is unicasted only to the selected path in the reverse direction. Destination also creates a shared session key between source and destination for transmitting secure data in future. To forward RREP in the reverse path, destination creates a random number, a nonce, and a pseudonym and also computes shared session key between the penultimate node, say *I*_*k*_, and itself to create the cipher text. The format of the Route Reply (RREP) to the source from destination is shown in

(13) RREP=NonceD,NymD,EKIkDRREP,EIDSIDS,IDD,NSR,snum.

Intermediate nodes forward this RREP to the source node by using the path stored in their routing table. The route that is obtained for data transmission through this phase is privacy preserved secure reliable route. Hence, data packets that flow in this reliable path are more secure and strong privacy protection is achieved for these packets.

#### 3.2.4. Unobservable Message Transmission

Unobservable message transmission is done after the above route discovery process. After the source receives RREP packet from the destination, it starts message transmission under the protection of secure keys. Source node sends the message to the destination through the discovered route as (Nonce_*S*_, Nym_*SI*1_, *E*_*K*_*SI*1__(MSG, snum, *E*_*K*_*SD*__(payload))), where MSG denotes the packet type (message). When the destination receives the message through the intermediate nodes available in the discovered secure reliable path, it decrypts it with the right key and obtains the original message. Now the packets transferred from source to destination are unobservable by intruders. Thus, an unobservable message transmission is achieved from source to destination.

## 4. Privacy and Security Analysis

### 4.1. Privacy Analysis

The proposed routing scheme provides anonymity, unobservability, and unlinkability properties to ensure the privacy by implementing group signature and ID-based encryption mechanisms in mesh routers for route discovery. The proposed protocol is implementing the improved version of the mechanisms as discussed in [10], and the privacy analysis of the proposed protocol is carried out in the same way.

#### 4.1.1. Anonymity

Most people would like to remain anonymous while roaming in WMNs for privacy reasons. In this proposed scheme, session key is generated between each node with its neighbors. By using this secret session key, each node can authenticate each other anonymously and subsequent route discovery process is built based on these session keys. Since pseudonyms and nonces are used in this route discovery process, the nodes present in the network cannot be able to reveal sender's identity.

#### 4.1.2. Unlinkability

In the proposed protocol, the packets (control packets and data packets) are identified by pseudonyms which are generated from random nonces and secret session keys. Within the RREQ message, except the nonce and pseudonyms, the remaining part of the message is encrypted and decrypted at each hop. Hence, for an attacker who can eavesdrop any transmission within the network, it is very difficult for him to find relation between the messages without knowing the encryption key.

#### 4.1.3. Unobservability

Since the nodes involved in the route discovery process of the proposed protocol are anonymous to other valid nodes, the control and data packets are indistinguishable from dummy packets and unobservable to the adversary nodes. It is very difficult for an attacker to find any relation between pseudonyms and nonces since nonces are updated periodically. Only the nodes with valid session keys will be able to decrypt and open the cipher text available within the message. Hence, the protocol ensures complete unobservability to the adversary nodes.

### 4.2. Security Analysis

It is known that most of the external and internal attacks against the routing protocols can be prevented by encryption and authentication mechanisms. The proposed scheme, in addition to the strong key management scheme, implements a new Cross-Layer and Subject Logic based Dynamic Reputation (CLSL-DR) mechanism to provide strong security against Denial of Service (DoS) attacks like packet dropping and misdirecting attacks, route disrupting attacks, and so forth. Compared to the existing routing mechanisms, the proposed protocol provides better security against the following DoS attacks.

#### 4.2.1. Packet Dropping and Misdirecting Attacks

PSRR is resistant against packet dropping and misdirecting attacks like worm hole, black hole, gray hole, and jellyfish attacks. The proposed protocol mitigates all these attacks, since the discovered route is completely privacy protected and trust level metric is implemented in the route discovery process which captures all the misbehavior information. As a result, the malicious nodes are isolated during route discovery. The trust level metric of each neighbor node is computed based on link quality metric MEFW which guarantees high performance reliable path. By introducing this efficient reputation computation mechanism in our proposed protocol, the protocol provides strong privacy and security protection against packet dropping and misdirecting attacks.

#### 4.2.2. Access Control

Our protocol ensures that only legitimate users can gain access to mesh networks. To be able to access the mesh network, each node has to satisfy the trust level requirements and also it has to obtain a group signature signing key and an ID-based private key through the key management scheme. An adversary cannot easily get these keys for proper authentication and also these nodes are isolated during route discovery. Hence, the access control to the network is provided.

#### 4.2.3. Preventing Route Disruption Attack

Route disruption attack is caused by the malicious behavior of a node through modification of a mutable field and dropping routing information elements. It may be noted that, in our scheme, only authenticated nodes can participate in the route discovery phase. Moreover, routing information elements are authenticated and verified per hop. So, it is not possible to launch a route disruption attack.

## 5. Implementation and Performance Analysis

The proposed PSRR protocol has been implemented and analyzed in the network simulator NS2. The proposed scheme utilizes a network topology comprising of 25 wireless mesh routers that provides communication to other networks. We evaluate the security capability of PSRR in terms of the parameters such as Packet Delivery Ratio (PDR), Route Acquisition Delay (RAD), average end-to-end delay, False Positive Rate (FPR), and message overheads. For privacy analysis, we have considered an entropy based privacy metric to analyze the anonymity of sender RREQ messages.

For the security analysis, we are comparing the proposed protocol with the traditional wireless mesh network protocol named Hybrid Wireless Mesh Protocol (HWMP) and similar privacy preserved protocol designed for wireless mesh networks named Privacy Aware Secure HWMP (PA-SHWMP) [4] in both malicious and nonmalicious environments. Privacy analysis of the proposed protocol is compared only with PA-SHWMP. Simulation experiment setup is shown in Table 1.

**Figure 2:**
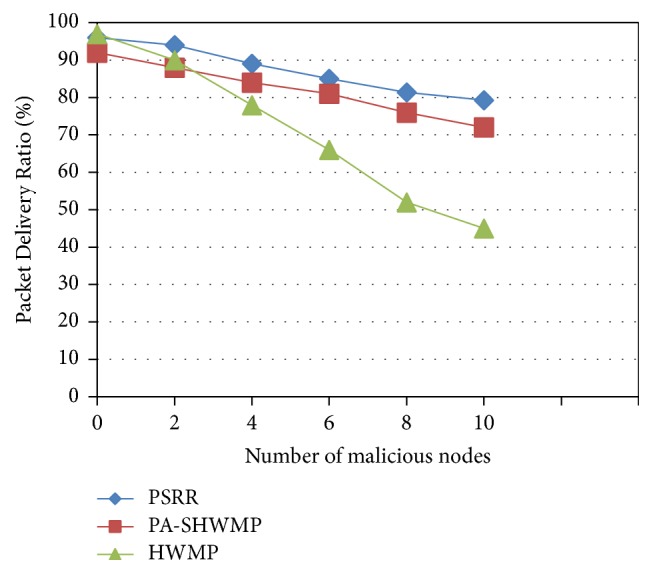
Packet Delivery Ratio versus number of malicious nodes.

Figure 2 shows the Packet Delivery Ratio analysis with respect to the number of malicious nodes present. This is the ratio of total number of packets successfully received by the destination nodes to the number of packets sent by the source nodes throughout the simulation. In addition to group signature and ID-based encryption scheme, PSRR implements CLSL-DR mechanism to select privacy preserved secure reliable route and the packets are forwarded only by the trusted intermediate nodes. In nonmalicious environment (zero malicious nodes), all the three protocols provide higher performance in PDR as shown in Figure 2. When the numbers of malicious nodes are increased, PSRR and PA-SHWMP provide better performance, whereas the PDR performance of HWMP is highly degraded. By implementing CLSL-DR mechanism in mesh routers, trust values are computed for each neighbor node by considering the reliability of the links and forwarding behavior of the nodes. In both the protocols PSRR and PA-SHWMP, malicious nodes are isolated during route discovery and message transmission. Compared to PA-SHWMP, PSRR selects the most reliable route in addition to privacy protection and security. Hence, the proposed protocol shows better performance compared to the other two protocols.

**Figure 3:**
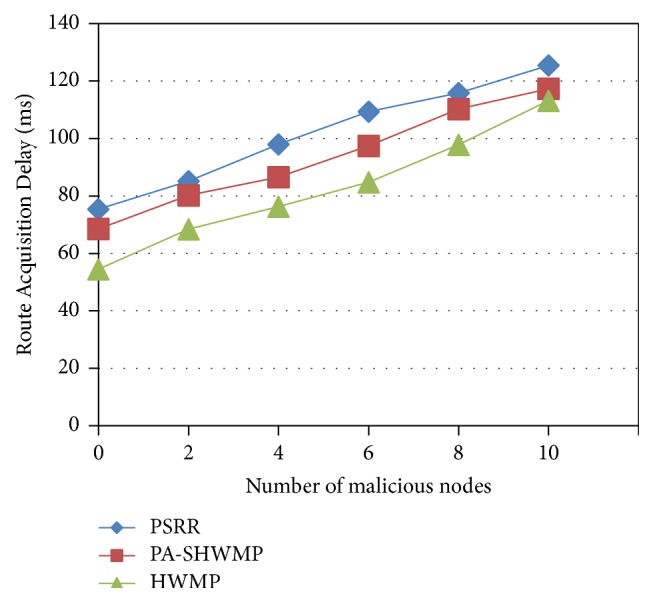
Route Acquisition Delay versus number of malicious nodes.

Figure 3 shows the Route Acquisition Delay (RAD) comparison of the proposed protocol and the other two protocols PA-SHWMP and HWMP. This metric is measured by computing the time interval between forwarding Route Request (RREQ) message to a destination and getting the Route Reply (RREP) at the source node. In this scenario, same source and destination nodes are kept and by varying the malicious nodes from 2 to 10, the delay values are computed and compared for all the three protocols. Even though the proposed protocol minimizes the control packets overhead, the computation time required for implementing privacy and security mechanisms is high. Comparing the three protocols, PSRR provides higher delay values by keeping the advantage of selecting the most reliable route.

**Figure 4:**
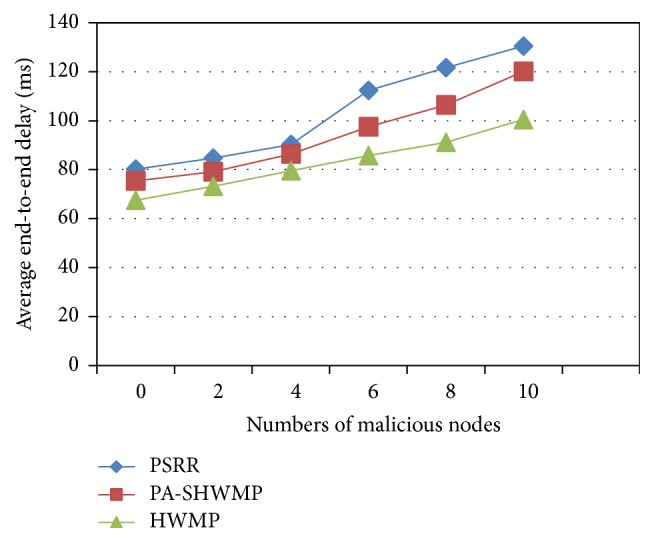
Average end-to-end delay versus number of malicious nodes.

Figure 4 shows the average end-to-end delay ratio of PSRR and a comparison with PA-SHWMP and HWMP. End-to-end delay is the time taken for a packet to reach a destination from a source. In both malicious and nonmalicious environments, PSRR gives higher end-to-end delay values than the other two protocols. This is because the proposed protocol implements CLSL-DR mechanism for reputation computation and reliable route discovery in addition to authentication and encryption mechanisms to have complete privacy protection and security. Hence, the computation required for the trust level metrics and cryptographic mechanisms make the end-to-end delay of the proposed protocol high compared to the other two protocols in both malicious and nonmalicious environments.

**Figure 5:**
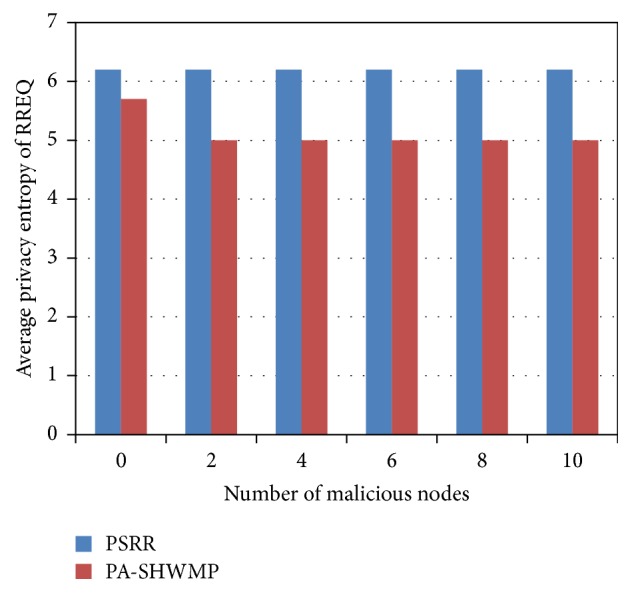
Average privacy entropy of RREQ versus number of malicious nodes.

For privacy analysis of the proposed protocol and the other similar privacy preserved routing protocol PA-SHWMP, we have considered an entropy-based privacy metric according to information theoretic approach as discussed in [10]. This metric is computed according to the probability distribution of a node being a sender. Here, we consider the sender anonymity of RREQ packets to measure the entropy metric for our analysis. It is computed as shown in

(14) $$\begin{array}{c} E_{k}=-\sum p_{j}\log_{2}\;p_{j}, \end{array}$$

where *p*_*j*_ is the probability of node *j* being the sender of RREQ_*k*_ packet. This metric gives the bits of information that the attacker should know to effectively identify the sender of RREQ_*k*_ packet. The comparison analysis of PSRR and PA-SHWMP based on entropy metric is shown in Figure 5. It is observed that PSRR provides better privacy protection compared to PA-SHWMP by analyzing the entropy metric of RREQ packets. Since the proposed protocol provides complete unobservability, unlinkability, and anonymity, the sender anonymity of RREQ packets is better, which in turn provides constant performance of entropy metric irrespective of the increase in the number of malicious nodes.

Next we analyze the False Positive Rate (FPR) analysis for the three protocols by varying the percentage of lossy links in the network. Normally, packet dropping in wireless networks may happen by intentional behavior of attackers or by poor link quality. FPR is computed as follows:

(15) $$\begin{array}{c} \text{FPR}=\frac{\text{Number of normal nodes falsely identified as malicious nodes}}{\text{Total number of normal nodes participated in the network}}. \end{array}$$

**Figure 6:**
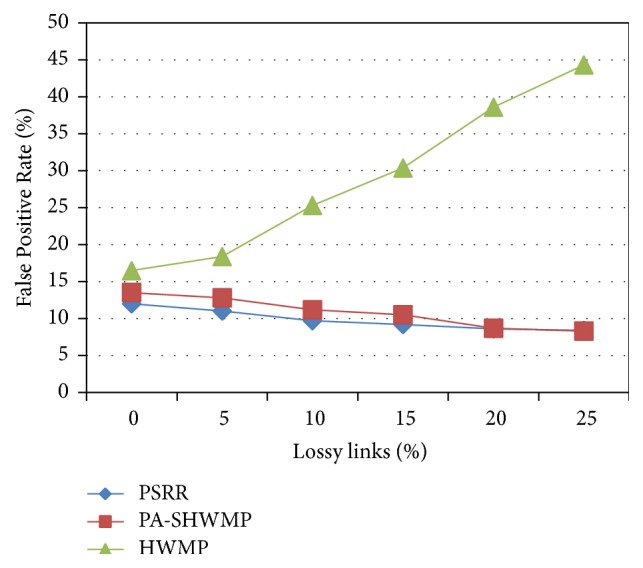
False Positive Rate versus percentage of lossy links.

Figure 6 shows that, among the three protocols, HWMP gives gradual increase in False Positive Rate when there is an increase in percentage of lossy links. This is due to the reason that the mechanisms for the differentiation between packet drops due to poor link quality or malicious nodes are absent in the HWMP protocol. The proposed protocol and PA-SHWMP give similar performance in FPR even in the case of increase in the number of lossy links. In the proposed protocol and PA-SHWMP, reputation, security, and privacy mechanisms are incorporated in the mesh routers and hence packet dropping with different reasons is correctly recognized which in turn decreases the False Positive Rate.

**Figure 7:**
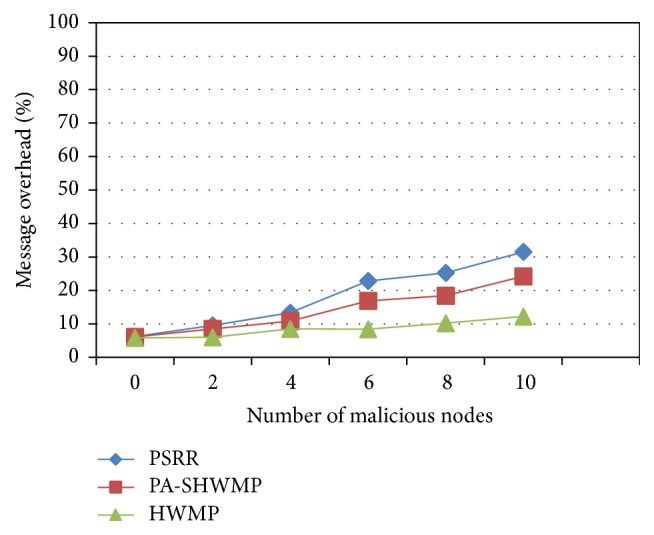
Message overhead versus number of malicious nodes.

Figure 7 shows the comparison analysis of message overhead for the proposed protocol and the other two protocols. The message overhead is less in HWMP since it does not include additional control messages required for either trust computation or for cross-layer message exchange other than the basic control messages that are required for route dicovery. The proposed protocol PSRR has additional message exchanges due to the implementation of trust computaion that involves the cross-layer exchange of link quality information. This cross-layer exchange of link quality information is required to improve the reliability of the discovered route. The message overhead involved in PA-SWHMP is in between the other two protocols since it focusses on privacy and security and does not provide reliability. There exists least difference in message overhead for the three protocols when the number of malicious nodes in the network is less. When the number of malicious nodes is increased, linear increase in message overhead is observed in PSRR and PA-SHWMP.

From the above analysis, it is clear that the proposed scheme (PSRR) provides better security and privacy performance than PA-SHWMP and HWMP by implementing a new CLSL-DR mechanism to compute trust values and link reliability in addition to the group signature and ID-based encryption schemes for preserving the privacy. However, the proposed protocol suffers in terms of end-to-end delay, Route Acquisition Delay, and message overhead parameters by keeping the advantage of providing better security, privacy, and efficient data transmission.

## 6. Conclusions

This paper deals with the design, implementation and a detailed analysis of Privacy preserved Secure Reliable Routing (PSRR) scheme in Wireless Mesh Networks. The design of PSRR offers strong privacy protection by satisfying the properties like anonymity, unlinkability, and unobservability completely. The design also includes the discovery of secure reliable routing path by employing CLSL-DR mechanism for efficient data transmission in wireless mesh networks. The privacy and security analysis demonstrate that PSRR is not only resistant to privacy related attacks but also resistant against the misbehavior of the malicious nodes caused by packet dropping and misdirecting attacks. After the implementation of three protocols in malicious and nonmalicious environments, it is observed that the proposed protocol provides better performance compared to PA-SHWMP and HWMP with respect to privacy and security analysis. The simulation results show that PSRR and PA-SHWMP provide more or less similar performance in terms of the parameters such as PDR, RAD, end-to-end delay, FPR, privacy based entropy metric, and message overhead. By implementing a new cross-layer based reputation mechanism in the proposed protocol PSRR, the link reliability and link quality are well focused which in turn gives higher PDR. Moreover, PSRR is able to keep the malicious nodes well isolated. Hence, the proposed protocol PSRR is privacy preserved and reliable and also provides more security against internal attacks in WMN. However, the proposed protocol suffers in terms of end-to-end delay, Route Acquisition Delay (RAD), and message overhead due to the implementation of cryptographic mechanisms, reputation metric computation, and cross-layer information exchange mechanism during route discovery.

In future, performance of the protocol can be enhanced further in terms of privacy and security by incorporating efficient cryptographic mechanisms and different perspectives in reputation computation such as Bayesian theorem and Game theory mechanisms and also to focus on minimizing the computation overhead in wireless mesh networks.

## Figures and Tables

**Table 1:** Simulation parameters.

Parameters	Values
Number of nodes	25
Total simulation time	50–250 s
Packet size	1000 bytes
MAC protocol	802.11n
Radio transmission range	250, 550 m
Area size	1000 m × 1000 m
Protocols	PSRR, PA-SHWMP, and HWMP

## Notations

*N*: A node in wireless mesh network
*P*: A big prime number
*S*: Master secret key owned by the key server
*R*: Generator of the elliptic curve group *G*1
*H*_*i*_(*∗*): Secure one-way hash functions, *i* = 1,2, 3
gsk_*N*_: Private group signature key of node *N*
gpk: Public group signature verification key
*K*_ID_*N*__: Node *N*'s private ID-based key
*E*_*N*_(*∗*): ID-based encryption using *N*'s public key
${\overset{-}{K}}_{N^{*}}$: A local broadcast key of node *N*
*K*_*NX*_: Shared session key between nodes *N* and *X*
Nym_*N*_: Pseudonym only valid within *N*'s neighborhood.

## References

[1] Akyildiz I. F., Wang X., Wang W. (2005). Wireless mesh networks: a survey. *Computer Networks*.

[2] Wan Z., Ren K., Zhu B., Preneel B., Gu M. (2010). Anonymous user communication for privacy protection in wireless metropolitan mesh networks. *IEEE Transactions on Vehicular Technology*.

[3] Lin H., Hu J., Ma J., Xu L., Nagar A. (2014). A role based privacy-aware secure routing protocol for wireless mesh networks. *Wireless Personal Communications*.

[4] Lin H., Ma J., Hu J., Yang K. (2012). PA-SHWMP: a privacy-aware secure hybrid wireless mesh protocol for IEEE 802.11s wireless mesh networks. *Eurasip Journal on Wireless Communications and Networking*.

[5] Mogaibel H. A., Othman M. Review of routing protocols and it's metrics for wireless mesh networks.

[6] Campista M. E. M., Esposito P. M., Moraes I. M. (2008). Routing metrics and protocols for wireless mesh networks. *IEEE Network*.

[7] Mahmoud M. M. E. A., Taha S., Misic J., Shen X. (2014). Lightweight privacy-preserving and secure communication protocol for hybrid Ad Hoc wireless networks. *IEEE Transactions on Parallel and Distributed Systems*.

[8] Ramya R., Navamani T. M., Yogesh P. Secured identity based routing and privacy preservation in wireless mesh networks.

[9] Mahmoud M. M. E. A., Lin X., Shen X. (2013). Secure and reliable routing protocols for heterogeneous multihop wireless networks. *IEEE Transactions on Parallel and Distributed Systems*.

[10] Wan Z., Ren K., Gu M. (2012). USOR: an unobservable secure on-demand routing protocol for mobile ad hoc networks. *IEEE Transactions on Wireless Communications*.

[11] Paris S., Nita-Rotaru C., Martignon F., Capone A. (2013). Cross-layer metrics for reliable routing in wireless mesh networks. *IEEE/ACM Transactions on Networking*.

[12] Khan S., Loo K.-K., Mast N., Naeem T. (2010). SRPM: secure routing protocol for IEEE 802.11 infrastructure based wireless mesh networks. *Journal of Network and Systems Management*.

[13] Khan S., Alrajeh N. A., Loo K.-K. (2012). Secure route selection in wireless mesh networks. *Computer Networks*.

[14] Bansal D., Sofat S., Singh G. Secure routing protocol for Hybrid Wireless Mesh Network (HWMN).

[15] You Z., Wang Y. (2012). An efficient and secure routing protocol for a hybrid wireless mesh network. *Journal of Computational Information Systems*.

[16] Khan S., Loo J. (2012). Cross layer secure and resource-aware on-demand routing protocol for hybrid wireless mesh networks. *Wireless Personal Communications*.

[17] Seth S., Gankotiya A. Denial of service attacks and detection methods in wireless mesh networks.

[18] Yu Y., Peng Y., Yu Y., Rao T. (2014). A new dynamic hierarchical reputation evaluation scheme for hybrid wireless mesh networks. *Computers and Electrical Engineering*.

[19] Sun J., Zhang C., Zhang Y., Fang Y. (2011). SAT: a security architecture achieving anonymity and traceability in wireless mesh networks. *IEEE Transactions on Dependable and Secure Computing*.

[20] Kathyaini N. A., Ananthakumaran S. Unconditional security based privacy protected user communication in Wireless Mesh Networks.

[21] Jeong I. R., Kwon J. O., Lee D. H. (2006). A Diffie-Hellman key exchange protocol without random oracles. *Cryptology and Network Security: 5th International Conference, CANS 2006, Suzhou, China, December 8–10, 2006. Proceedings*.

